# Editorial: T Cell Alterations in Adipose Tissue During Obesity, HIV, and Cancer

**DOI:** 10.3389/fimmu.2019.01190

**Published:** 2019-05-28

**Authors:** Dorothy E. Lewis, Joanne Lysaght, Huaizhu Wu

**Affiliations:** ^1^McGovern Medical School, The University of Texas Health Science Center, Houston, TX, United States; ^2^Department of Surgery, Trinity Translational Medicine Institute St. James's Hospital, Dublin, Ireland; ^3^Section of Cardiovascular Research, Department of Medicine, Baylor College of Medicine, Houston, TX, United States

**Keywords:** T cells, obesity, cancer, HIV, inflammation

This collection of articles is designed to foster understanding of the variety and function of T cells in adipose from different species in different disease states. This work originally developed from the obesity epidemic. As the obesity epidemic grew, initial work recognized that adipose was dysfunctional. Work focused on inflammatory macrophages that increase in adipose during over-nutrition to produce inflammatory cytokines (1–3), which also serve to increase the dysfunction of adipose. However, it was soon realized especially in mouse studies that T cells had an increased role, specifically that obesity was associated with an increase in Th1 and Th17 T cells that make inflammatory cytokines (4–9), which also drive adipose dysfunction (4, 10, 11). Recent studies suggest that obesity in cancer patients may be a major risk factor for acquisition Lauby-Secretan et al. (12) but a positive feature in response to certain therapies for certain tumors Wang et al. (13). Because there is widespread adipose dysfunction in HIV infected people, there was also focus on how and why adipose changed, whether it was in response to antiretroviral therapy or due to infection itself (14, 15). This collection of articles are mainly reviews which cover what is known about T cells in obesity, in cancer, and in HIV.

Two articles Wang and Wu, Zhou and Liu and examine the role of T cells in metabolism as well as the interactions with antigen presenting cells in adipose. The review by Wang integrates what we know about T cells and macrophages in adipose and how they function and differ in obesity vs. the lean state. The Liu review nicely shows what happens after over feeding as the cells and resulting molecular consequences in adipose tissue changes. There is an initial response by adipocytes in the first days, followed by induction of pro-inflammatory cytokines and recruitment of T cells and macrophages in the first 2–3 weeks, followed by reduction in Foxp3 at 12 weeks in the mouse. How this develops in humans is not clear, although constant high fat feeding takes very little time to cause major changes in appearance and lipid and liver enzymes as seen in the movie, Super Size Me released in 2004. In the movie, Morgan Spurlock ate only at McDonald's for a month. He gained 11 lbs that took him 14 months to lose.

Another article examines how and why the T cells in aging animals, which are also usually obese, might be different than those found in young obese animals Antony et al. In particular, the differences seem to lie in differences in T reg numbers or function in aging. In particular the numbers of T regs increase in aging and their function changes perhaps as a response to the prolonged lipophilic environment found in aging mice. Work on human adipose in aging remains to be done, but from mouse studies it is clear that the changes in adipose are different in aging than in mere obesity.

Two articles focus on the role of T regs in regulation of metabolism in obesity and in different species. The review by Zeng et al. covers what is known about the function of T regs in adipose in humans and mice, noting that several studies in humans associated Foxp3 expression with obesity. However, measuring T regs by flow cytometry has produced inconsistent results. There is some concern that variations might be due to fresh vs. frozen tissue observations, since T reg staining is reduced by freezing in some cases. In addition, human CD4 T cells express Foxp3 when they are activated, it is normally transient, therefore results of Foxp3 mRNA studies are confounded by that caveat. Cold also induces T regs in mouse adipose, but it is unknown if the same is true in humans. Finally, the authors present a nice summary of attempts to exploit T regs for obesity therapy. A comparison of adipose from different species (humans, mice, and cynomolgus monkeys) was done by Laparra et al. which clearly indicates that most animals except for the C57 mouse do NOT have increased Foxp3 T cells in the lean state. Adipocyte size is also markedly different between the three species. The dramatic conclusion is that mouse and human adipose tissue have markedly different immune cell compositions which should offer caution when interpreting mouse studies. More studies need to be done on young humans and on feral mouse populations to know whether environmental differences could contribute to these differences.

Two articles concern characteristics of T cells in cancer patients. One (Cornò) examines the information about innate lymphocytes in adipose and how they differ in obesity, as these cells likely act as sentinels to control tumor development Del Corno et al. Most of the work has been done in the peripheral blood and indicates that there is a decrease in many innate lymphocytes in the blood in obesity. In adipose there is some evidence for a decrease but more work is needed to directly link these cells to development of cancer. Another paper Conroy et al. reports novel data for the chemokine fractalkine found in omentum as well as its receptor (CX3CR1) in cancer patients and links it to memory CD8 T cell recruitment to the omentum perhaps bypassing the tumor and leading to a mechanism whereby tumor cells are allowed to go unchecked.

A single article reviews human T cells found in HIV and compares the information to the T cell changes in obesity Wanjalla et al. A key difference in HIV is that there are clonal populations of CD8 T cells and those cells predominate with reduced CD4 T cells. In obesity, although CD8 T cells have greater increases in adipose compared to lean (6, 16) there are higher levels of polyclonal memory CD4 T cells than CD8 T cells (16). In addition, in HIV there are fewer Tregs, with more in obese humans. Finally a key recent observation is that CD4 T cells in human adipose express more PD-1 which has implications for adipose as a reservoir for HIV.

In summary, this collection of articles provide the background to focus efforts on preventing the consequences of adipose dysfunction seen in so many disease states.

## Author Contributions

All authors listed have made a substantial, direct and intellectual contribution to the work, and approved it for publication.

### Conflict of Interest Statement

The authors declare that the research was conducted in the absence of any commercial or financial relationships that could be construed as a potential conflict of interest.
